# Report of the Onondaga County Medical Society, on "Mineral Paste."

**Published:** 1844-03

**Authors:** A. Westcott

**Affiliations:** Syracuse, N. Y.


### ARTICLE VII.

### *Report of the Onondaga County Medical Society, on "Mineral Paste."* By A. WESTCOTT, M. D. of Syracuse, N. Y.

SYRACUSE, *Feb.* 1, 1844.

*To the New York Editor :—*

MY DEAR SIR:—Agreeably to your request, I embrace the earliest opportunity to forward you the report of the committee appointed by the Onondaga County Medical Society, to investigate the merits of "mineral paste," as an article for filling teeth. I transmit, also, some other facts and considerations, relating to this subject, which should you regard of sufficient interest, you may insert in the forthcoming number of the Journal. It may be deemed by those from whose territory this pernicious article has been already banished, by the persevering effort of a few individuals, too late in the day, to write exposes of this species of quackery. I would it were so in this region, but when I assure you that not more than a year since, at least one-half of all the teeth filled in this village, (containing about eight thousand inhabitants,) were filled with *mineral paste*, you may suppose that all the *dental trowels* are not yet gone into disuse. Nor has it been *itinerant* quacks alone, who have presumed to employ it and advocate its virtues.

About one year ago I caused to be published in one of our village papers, an article designed as an expose of this branch of empiricism, I will quote so much of that article, as is not already published in the Dental Journal, it is as follows:

DENTAL QUACKERY—Messrs. Editors—Dear Sirs:—Permit me through your columns to present to the public, the following expose.

Every practice either in medicine or surgery, if it be pernicious in its ultimate results, is the more dangerous to the community, as it is the more alluring, and of the many which are offered to the public, perhaps few so destitute of merit, present more apparent advantages, than that of filling teeth with "mineral paste," especially to those who are ignorant of its composition, and tendencies. Indeed, who would not be induced to have their teeth filled when they are made to believe, that the operation may be performed "without pain" with little expense, and all this too with "*royal mineral succedaneum,*" which, though a paste, when introduced, soon "becomes as hard as the tooth itself!" Though this paste has at different times, received different appellations, changing with the fancy or policy of the operator, yet its composition has ever been the same. It uniformly contains as an *essential,* nay, as the *chief* ingredient, *mercury* or quicksilver, the other may be silver, tin or zinc.

From the mercury of this compound when oxydized or corroded, as it always is by the fluids of the mouth, there is formed a substance closely allied to *calomel,* indeed, judging from its *effects,* we might say it more nearly resembled corrosive sublimate which is but another compound of mercury. The above facts will give an easy solution to the phenomena, described in the following letter from Dr. J. Stearns, dated,

POMPEY, *Dec.* 15, 1842.

DEAR SIR:—Regarding as I do the profession of dentistry as a science, which ought to be ranked among the honorable professions, I therefore feel it my duty for the public good, and the honor of your laudable profession, to expose the vile quackery that is being practised on the community in this section, by an *itinerant* dentist, to the great injury of many individuals, and the success of the practice of dentistry. I will here give a statement of a case or two, which have fallen under my observation. Miss R. S. called me to visit her. I found her with febrile symptoms; her tongue, gums and glands swollen; a free discharge of saliva; a fetid breath, &c. I asked her if she had ever been salivated—she said never, I was positive, however, that she was under the influence of mercury, and then found that two or three weeks before, she had several teeth filled with "*royal mineral succedaneum.*" The teeth were very loose; the next day I removed one of the teeth, found it perfectly dead, and the alveolar process affected, which I have since removed, including almost the entire socket of the tooth. I have since been called to Mrs. W. who has been *severely salivated* by the use of the same compound.

Respectfully, yours, JEHIEL STEARNS.

A. WESTCOTT, M. D.

It is proper here to remark, that the effect as described by Dr. S. in the above letter, is not the same in all cases. I have seen several cases within the last year, nearly as strongly marked, yet perhaps with a majority of those who have had teeth filled with this article, the effect does not amount to salivation in the common acceptation of that term. This much, however, may be safely said, and is of *universal* application, that its tendency in this respect is *always* bad, and in *no* case where any considerable amount of it is used, does it fail to vitiate the secretions of the mouth to a greater or less extent. The *influence* it is capable of exerting, and *time* elapsing before it is rendered perceptible, will depend on the following circumstances,

1st. The susceptibility of the individual to the effects of mercury, it being well known that while some others are badly salivated by comparatively a trivial amount.

2d. The quantity of cement used.

3d. Age and health of the subject.

The remainder was made up of quotations from the different authors who had written on this topic in the Journal and the report of the committee of American Society, July, 1841. No sooner had this article made its appearance than it was construed by the oldest dental practitioner in this village, (Dr. Bliss,) into a personal assault, and that too aimed exclusively at himself! He sought redress by applying to the County Medical Society, of which he *claimed* to be a member, and entreated its interference in his behalf, pledging himself moreover that he would convince them that the cement *he used* was not only efficient in arresting caries, but entirely harmless. The society very reluctantly recognised the subject of complaint as coming within its province, but at length, as the easiest method of disposing of it, appointed a committee of three, to whom the investigation was referred, and who were to report at the next meeting of the society. I was soon after summoned by this committee to appear before them, and answer the arguments, which Dr. B. had pledged himself to give in support of his grand dental catholicon.

At the meeting of the American Society of Dental Surgeons, convened at Baltimore, in July last, I brought this subject up for consideration, asking such action upon it as might be deemed proper. This request was responded to by the following resolution, which was unanimously passed,

On motion of Dr. Harris, *Resolved,* That this society regards the use of mineral paste in plugging carious teeth as *malpractice,* and that a committee of three be appointed to receive information and facts on that subject to be transmitted to Dr. Westcott of Syracuse, in the state of New York, to be by him laid before the Medical Society of the county of Onondaga, in that state, before which the subject aforesaid is now pending.

This report was duly made, by the able gentleman to whom it was referred, and laid before the committee of the Onondaga Medical Society which, together with the report from that society, I place at your disposal. So much for the history.

On the discussion of this subject, which has been done frequently and efficiently, in the Journal, and elsewhere, the chief arguments against its use have been founded on its pernicious tendency in vitiating the fluids of the mouth, producing salivation, &c. Though *this,* independent of all other objections, is sufficient to condemn its use, there are other considerations which have not been fully weighed. I propose to offer some suggestions under the following heads in their order :

1st. It is uniformly and necessarily inefficient in arresting caries.

2d. It is in every case dangerous.

3d. It is destructive to gold in the mouth either in the form of fillings or plate.

4th. It is never called for.

*First.* Inefficiency, no question is now more clearly settled than that dental caries, is chiefly, if not wholly owing to the chemical action of the acrid fluids upon the calcareous portion of the tooth, and hence the only object gained by any filling, is the protection of the exposed bone from such attack. It follows, therefore, that any attempt failing to accomplish this end, must be futile. Is this end attained by amalgam fillings? I answer promptly, *no.* Before giving any special attention to this subject, that such fillings did not hinder ingress to fluids into the cavity. The first was from observing that the plug when removed, appeared *in all cases* much more thoroughly oxydized on its posterior, than on its anterior surface.

And second, in all cases where the filling had remained any considerable length of time, the tooth was soft behind it, its earthy matter having been absorbed; to put this question to the test, I instituted the following simple experiment:

After preparing a quantity of the amalgam very carefully, I filled with it a strong glass tube, making it as compact as possible, and then suffered it to congeal undisturbed, after which I immersed it in a tincture of red sanders. In a very few hours the colored fluid penetrated entirely around it, absolutely hiding the cement from view. Now if this was the result in the case just described, and that too before any oxydation of its sides had taken place, what must be the result in the tooth, where the advantages for perfectly packing must be much less than in the tube. I may be permitted here to state, that, among the evidences furnished by Dr. B. to the committee upon this subject was a tooth which contained a very large cement filling; this was immersed in the same fluid. On breaking the tooth, the coloring matter was found covering the entire surface of the filling. This point being an important one to settle definitely, both on account of its rendering these fillings inefficient, and also giving a much greater oxydating surface, I was induced to test its specific gravity as corroborating evidence, which alone would have settled clearly this point; upon this subject I need not repeat my own experiments, as we have ample testimony from good authority. In a treatise on chemistry by Wm. Henry, M. D., F. R. S., with additions by Prof. Silliman, in speaking of alloys, he makes the following remarks:

"The specific gravity of an alloy is seldom the mean of its component parts * * * An alloy of *silver with mercury*, though the former metal is specifically lighter than the latter, possesses so much acquired density as to sink in quicksilver." Now, if the above observation be correct, the actual shrinkage in the process of hardening cannot be less than two twenty-fifths.

To make the absurdity apparent of using the compound, with the hope of arresting decay in teeth, no comment is needed.

*Second.* It is, in every case, dangerous.

In order to substantiate this position, it is by no means necessary to prove that this compound does, in every instance, produce salivation; indeed, so far from this, if it can be shown that a single case has ever occurred from its use, the point is gained.

This conclusion rests upon the fact that *no man*, however skilful, can judge, a priori, either of the natural or acquired susceptibility of an individual, in respect to the effect of mercury upon the system. Hence, whoever places this amalgam in the mouth, does so at the *risk* of the worst consequences, which are ever liable to result. This, I say, would be true, had there occurred but a single case of the kind. But since we have ample testimony of salivation being a very common result, and, moreover, that a vitiation of the secretions of the mouth a uniform one, the *culpableness* of this practice stands out in bold relief. Next let us lay aside, for a moment, the evidence founded on an observation of the *result*, and examine the properties of this paste, to see whether, by such examination, we should be led to anticipate what observation has shown to be true. To put this to the test, the only conditions necessary to be established are,

1st. That the mercury of the amalgam would become oxydized, and,

2d. That the amount of oxyd formed would, either as oxyd or as salts, by combination with the various acids with which it is liable to be brought in contact in the mouth or stomach, produce salivation.

Under what circumstances, then, is mercury oxydized?

"Mercury is oxydized by agitation in a bottle, half filled with air, and is converted into a black powder."*

Now it is clear that any metal which would be thus affected by simple contact of air, would be far more liable to oxydation in the mouth. For example, tin and silver will retain a *polish* when exposed to *air* and *moisture;* but either of them is very soon corroded in the mouth.

The next question to settle is, whether this susceptibility of oxydation is enhanced by its union with silver as an amalgam? The following testimony, from Dr. Turner, is in point:

"The tendency of metals to unite with oxygen is considerably augmented by being alloyed.† This effect is particularly conspi-

* Henry's Chemistry, page 40.

† *Alloy* is a generic term, and includes all the various combinations formed by the union of one metal with another, while the amalgam is specific, and applies only to such metallic compounds as have mercury as one of the constituents.

cuous when dense metals are liquified by combination with quicksilver. Lead and tin, for instance, when united with mercury, are soon oxydized by exposure to the atmosphere, and even *gold* and *silver* combine with oxygen, when the amalgams of these metals are agitated with air! The oxydability of one metal in an alloy appears, in some instances, to be increased in consequence of galvanic action. Thus, Mr. Faraday observes, that an alloy of steel with one-hundredth of its weight of platinum, was dissolved with effervescence in diluted sulphuric acid, which was so weak that it scarcely acted on common steel; an effect which he ascribed to the steel in the alloy being rendered positive by the presence of the platinum.*

If a similar galvanic action results from the union of silver and mercury in mineral paste, the mercury would, of course, bear the relation to the silver as did the steel to the platinum, in the case cited by Mr. Faraday. These two metals, together with the fluids of the mouth, would constitute all the essentials to a galvanic pile. If we add to these considerations the fact, that while "the current formed by the contact of two metals, gives increased effect to the affinity of one of them for some element of the solution, the ability of the other metal to undergo the same change is proportionally diminished," we have at once a clear idea of the changes which occur, and the successive steps in the process. By the galvanic agency exerted betwen these two metals by the fluids of the mouth, the natural affinity of the amalgam for oxygen is so increased as to make the decomposition comparatively rapid. But the most important conclusion which may be drawn from these premises, is the obvious fact, that the mercury (the most oxydable metal) would undergo much the most important, if not the entire change.† For "when plates of zinc and copper touch each other in dilute acid, the zinc oxydizes *more,* and the copper *less,* rapidly than without contact." On the same principle, precisely, is the silver of this compound protected at the expense of the mercury. It follows, hence, that while the presence of the silver greatly facilitates the oxydation of the mercury, the

* See Turner's Elements of Chemistry, 6th Am. ed., p. 399.

† See Turner, p. 89.

latter metal is principally acted upon, and the result must be a copious formation of a mercurial oxyd, corresponding to the amount shown by observation to be formed.

The only remaining question is as to whether the amount thus formed is capable of affecting the system in the way complained of. Of this, after presenting the following facts and considerations, I leave all to judge, who will lay aside prejudice. The amount of calomel, and other preparations of mercury, required to produce salivation, may be seen from the following quotations from the London Lancet, and the British Foreign Medical Review:

"Very lately, a man who was taking sulphuric acid for epistaxis, was severely salivated by two grains of calomel, in a dose of cathartic pills. *The acids naturally contained in the stomach were the muriatic and acetic, and the mercury contained in two grains of calomel, or a few grains of blue pill, would of course be sufficient to produce the most serious consequences, if changed into the bi-chloride, or corrosive sublimate.*"—DR. SNOW.

The following is the testimony of Mr. Streeter:

"The fact of the action of blue pill being much more powerful at one time than another, might be accounted for by the conserve of roses, with which the mercury was triturated, being occasionally mixed with sulphuric acid, for the purpose of restoring its lost color. Hence, instead of the simple oxyd, the sulphate of mercury was producing its effect on the patient. He had no doubt, however, that some constitutions were remarkably susceptible of the influence of mercury in its mildest forms. He had seen severe salivation produced by the administration of nine grains of blue pill, although it had been given in only *one grain doses*, three *times* a day, and its effects watched with the greatest care."*

We give the following quotation from Dr. Law, in corroboration of the above:

"We directed *one* grain of calomel to be mixed up with a sufficient quantity of extract of gentian to make a mass, to be divided into twelve pills, one of which was to be taken every hour. We found, in some cases, salivation to be produced by twenty-four of these pills, or two grains of calomel, and seldom were forty-eight pills, or four grains, required to produce this effect. * * * * * We exhibited the blue pill in the same way, and found the mouth to

* London Lancet, Jan. 18, 1840, p. 625.

become sore from six grains.† * * * * We have found one drachm in every night sufficient to produce salivation."‡

Dr. Law accounts for salivation by such minute doses of mercury, on the supposition that a succession of impressions is thus kept up by the frequency of their administration. Nothing is more clear than that a very small quantity of mercury is capable of producing salivation, much less, I propose to show, than would result from a single cement filling.

I have one of these fillings in my possession which weighs sixty-three grains. Now this amount of amalgam would be sufficient to produce from the mercury it contains, thirty-seven grains of calomel, and about thirty-eight and one-half grains of corrosive sublimate. On the supposition that the *whole* of this was administered as calomel, in the way described above, it would be more than sufficient to salivate eighteen persons!

What are the real facts in the case? It has been shown that by the contraction of the mass in the act of congelation oxydizing fluids, have free access to the *entire* surface of the filling. A large oxydating surface, is hence constantly exposed to the perpetual action of these fluids. This oxyd, while it remains such, would get into the system only from the outer surface of the filling, and hence it accumulates, and would continue to do so till either some extrinsic acid, or the acid of the saliva, which frequently occurs under peculiar states of the system, unite with this oxyd, and convert the whole into a salt of mercury. It is a fact, moreover, that the mildest salt of mercury, which could be formed under any circumstances, is calomel, and inasmuch as the principal acids, prevailing both in the fluids of the stomach, and mouth are muriatic, with a small quantity of acetic, the presumption is, that corrosive sublimate, would be quite as likely to be the result, as calomel. Not only so, the mode of administration in respect to succession of doses, corresponds almost exactly with that above pointed out, in order to produce salivation with the least possible quantity of mercury. But while it is true, that in most instances, the mercurial action would thus be got up only at in-

† It will be recollected that the blue pill is essentially the same as the oxyd formed in the mouth before acted upon by acids.

‡ British and Foreign Review, April, 1840, p. 582.

tervals, alternating with changes of health, and natural fluids of the mouth, it is also true, that in many mouths, this oxyd of mercury, would be constantly changing into salts, from the uniform acidity of the saliva, and hence such persons would be constantly receiving the impressions from the oxyd of the entire surface of all the fillings in the teeth, and in *no case* would a person be freed from the amount of constant effect, which would follow from that portion of oxyd which accumulated on the outer surface of the filling. From the above facts and considerations, we make the following deductions :

1st. That every person wearing in the teeth any considerable quantity of this cement, is *liable* to salivation, from the accumulation of the oxyd shown above to take place.

2d. Every person is subject to so much of the *constant* effects, as would accrue from all the oxyd formed on the outer surface of these fillings, and which would be easily carried into the stomach.

3d. Those persons whose saliva is very generally more or less acid, would be subject to the *constant* impressions from the *entire oxyd* formed on the *whole* surface. In either of the last two cases, the effect would be sufficient to keep up an unhealthy action of the secretions of the mouth, if it did not amount to salivation.

4th. The constant oxydation and solution of the surfaces of these fillings, renders them less and less perfect, in respect to preserving the tooth, by giving more free access to those fluids which are the chief sources of decay.

*Third.* This amalgam is destructive to gold fillings and plate. The strong affinity of gold for mercury, renders this amalgam destructive to gold fillings or plate. I have seen in several instances the teeth which were clasped to secure the plate, filled with mercurial paste, and in each instance the clasp was literally eaten off at this point. To demonstrate this effect, we need only rub a piece of gold with the amalgam, when the surface will immediately become white to the extent that the amalgam of gold is formed. This amalgam like that of silver is oxydable and easily removed; gold plugs and plate are not only subject to this destructive process, from actual contact of the amalgam in its primitive form, but several salts formed from the oxydation of these cement fillings, and which are held in solution by the saliva, are also destructive to gold.

The fourth position we took in relation to this amalgam, was that its use is never called for. Though this position rests mainly on the ground, both of its inefficiency and hurtful tendency, yet were each of these qualities susceptible of remedy, it might easily be dispensed with. In substantiating this ground, we will briefly analyze the claims to confidence, which are set forth by its advocates, and which are offered as excuses for using it, the principal are the following:

1st. "Teeth so frail as cannot be filled with gold, may be filled with this amalgam."

2d. "A filling of this paste may be made to remain permanently, where one of gold could not."

3d. "The operation causes little or no pain," and

4th. "It is less expensive to the patient."

We remark in relation to the above reasons for employing "metallic cement," that they are so plausible without a careful examination, and such an one as few are enabled to give this subject, that its allurements are so strong, that we by no means wonder that so many unsuspecting people, have consented to have their teeth ruined with this worse than quack remedy, which unexamined, promised fair to shield them from one of the greatest ills which, at least *bone*, "is heir to."

It may be remarked also, that its claim is enhanced, not unfrequently by the former standing of those who recommend it; were its use confined to *itinerant* quacks, the case would be different, but when we find for example, a man who claims to have practised dentistry in the same village for fifteen years, and that he has read and practised medicine for several years; when we find such a man, though stooping to this low degree of empiricism, claiming that he has *successfully* filled within the last twenty-two months, at least three hundred teeth with this mercurial paste, we need not be astonished at the number who would be thus inclined to try its virtues. But we will examine each of the above asserted virtues of this paste.

1st. What is the fact in relation to saving by its use, these "frail teeth" referred to, or inasmuch as it has been clearly shown that the paste is incapable of saving *any* tooth, we may inquire more properly whether gold cannot be used in all teeth, capable under any circumstances of being successfully filled? If we are allowed to introduce testimony on this subject, we have it amply given in a resolution of the American Society of Dental Surgeons, in a convention held at Philadelphia, August, 1841. In this resolution, unanimously passed by said society, it is declared, that "there is no tooth in which caries in it may be arrested, and the organ rendered serviceable by being filled, in which gold cannot be employed."

It is well understood by all who are conversant with dental operations, that caries frequently occurs where it is impossible to retain the *entire shape* of the tooth, but I would inquire, what would be gained by either making up for *lost* substance, with an article which can serve only to *disfigure*, or by undertaking to fill *beneath* a portion of enamel, which would be fractured with the slightest pressure, with a material entirely incapable of preserving such delicate portion, or indeed any other part of the tooth. The fact is, that almost any tooth in which the nerve is not actually exposed, may be filled by a sufficient loss of substance, properly to shape the cavity. Under what circumstances then is it objectionable to sacrifice *any* of the substance of a tooth? The *necessary* loss would be almost exclusively confined to front teeth, and here the question again arises, what can be gained by filling under a portion of transparent frail enamel, with a substance which by its immediate oxydation and consequent discoloration, can only serve to disfigure, infinitely more than the actual loss of that portion of the teeth—to say nothing of the inefficiency or evil tendency of such fillings. On the supposition that this paste was not objectionable in itself considered, its use would very rarely be indicated for *such* reasons, in the hand of a skilful operator, who uses instruments of sufficient delicacy, and foil of a proper pliancy.

I venture to assert that an exception to the rule, that gold may be *generally* used, would not occur in one case of a thousand, and in *this* case, *paste* could not be made to stay. Query—would three hundred such cases occur in twenty-two months to a person in a very sparse practice?

It is asserted, 2dly, that cement can be made to stay, where gold could not. This assertion shaped a little differently, is doubtless literally true. In other words it is *doubtless* true, that *those who employ cement* could make *it* more permanent, at least in many instances, than *they* could, a gold filling. Were *I* under the necessity of charging my patients not to eat molasses candy, I certainly would resort to the use of cement, or abandon my profession. But the question is not whether gold would remain in the very cavity which some *tooth mason* had prepared for his *cement mortar*, but whether in any case, the cavity in question, might not, if skilfully prepared, be filled successfully with gold. This question is virtually answered by the American Society, in the resolution above quoted, where it is declared in substance, that *gold may be employed* in all those cases where it would be serviceable to fill a tooth, and so far as my own opinion can serve to substantiate this view of the subject, I give it without qualification, in concurrence with the society, as expressed in said resolution.

It is a very common impression by those who are unacquainted with the pathology of caries, that while a filling remains, the tooth is safe, but all who are at all conversant with this subject, know that this is far from being necessarily the case. I have often been called upon to extract a tooth, having in the very cavity which was the origin of the difficulty, a *gold* plug, which from the imperfect manner in which the operation was performed, had not arrested the caries, but which had been retained there, in consequence of the aperture through which it was introduced, being much smaller than the cavity within. This remark is particularly applicable to cavities filled with cement. That these fillings frequently remain till the mass by oxydation becomes much lessened, or till the tooth requires extraction from the ill effect of the paste, or from exposure of the nerve, I freely grant. The difficulty often is in removing them, and indeed this is the very thing complained of. Did they easily escape, it would break the charm, and fewer evils would result from this species of quackery. 3d. It is asserted, that the operation of filling with this paste, causes "little or no pain." In respect to this point, it is quite as well known to *patients*, as the operator, that the pain of the operation, is almost exclusively confined to the preparation of the cavity for receiving the filling, and if it is essential, as it certainly is, to remove entirely the carious portion, when the cavity is to be filled with *gold,* I would inquire, what makes it less essential, when it is to be filled with paste—and if the care in this respect, be the same in both cases, how can the operation be less painful? The only conclusion which can be drawn in relation to those operators who verify this promise to their patients, is, that *they do not remove the diseased portion of bone,* which no one denies to be essential. Few men have ever made a greater figure with mineral paste, than the famous or "infamous" Mons. Mallan, and in relation to him, *it is a fact,* that he caused *no pain* in the operation, and the secret lay behind the filling, which on being removed, the mystery was perfectly apparent.

It was, that not even the *foreign matter,* was removed. In a well attested case it was found, that on removing several of these fillings, immediately after they had been inserted, the cavities were partially filled with "pound cake," which had been purposely lodged there, but a few minutes before.

In relation to the *cheapness* of such fillings to those wearing them, I apprehend that little need be said, after demonstrating both their inefficiency and hurtful tendency. The idea of paying half the amount of a good article, for one much worse than nothing, is a policy easily understood. But this by no means is the extent of the evil. The money paid for the operation is not merely lost, but the *teeth* are thus sacrificed while the patient is feeling that they are being saved. Add to this the danger, and perhaps the actual occurrence of salivation, a siege of the tooth-ache, and finally the extraction and loss of the tooth, and we have a fair representation of the cheapness of a *very dear* operation.

Having now analyzed the qualities which it is claimed by its advocates to possess, I beg leave to offer an opinion, at least, upon the *real* inducements, or rather *temptations,* in all cases where the operator is not *culpably ignorant* of its real character, to employing this compound for stopping carious teeth. The principal are the following: 1st. It requires much less skill. 2d. It requires much less time, hard labor, and perplexity. 3d. The expense to the dentist is comparatively trifling.

That no special skill is required in using this article, hardly needs a single comment to prove. The fact that it becomes hard would render it evident, on the least reflection, that it would not be easily removed, in any case where the inner portion of the cavity was much larger than the outer. If this was not true of the entire cavity, if there were only two opposite projecting points, the object might be gained for a time. But, as has already been suggested, though it might remain, the tooth would not be benefitted unless it excluded from the cavity the fluids of the mouth; and the skill required to perform the operation would be far less than that exercised by the mason in making mortar adhere to an upper ceiling.

In respect to the comparative *time* required, we will let one of the chiefs of this tribe of operators speak for himself, in his own language, as is sworn to by several respectable witnesses:—"I can fill a tooth in a few seconds, sir, without giving the least pain. I filled thirteen teeth, yesterday, for a young gentleman of this city, a solicitor in chancery, a very liberal young gentleman. We can put in this composition of ours in *less than no time;* I filled those thirteen teeth yesterday in less than five minutes."

Here Monsieur Mallan asserts that he filled thirteen teeth in less than five minutes, whereas it not unfrequently happens that the most skilful operators spend *an entire day on a single gold filling*, meeting, at every turn, such difficulties and perplexities as can be appreciated only by those who have met and contended with similar ones.

In relation to comparative expense to the *dentist,* the sum is very easily stated. On the supposition that this cement contains sixty-four parts of mercury to thirty-six of silver, and estimating the mercury at one dollar per pound, and the silver at twelve dollars per pound, an ounce of this cement would cost forty-one cents, whereas an ounce of gold cost not less than thirty dollars, or more than seventy-three times as much as the same weight of mineral paste. To say the least, this would be a convenient saving, could it be made safely.

Having now briefly reviewed both the real and alleged properties of this paste, I subjoin the report of the committee appointed by the Onondaga County Medical Society, in June last. The circumstances giving rise to this committee, have been sufficiently detailed in the former part of this article; it will be borne in mind that it was appointed at Dr. B's earnest solicitation.

The report is as follows, viz.

The Committee to whom was assigned the duty of reporting to this Society upon the utility and safety of the royal succedaneum, in reference to its use in filling decayed teeth,

Observe—that there is a difficulty appertaining to the investigation of the subject, arising from the fact that the more intricate dental operations have been but in a limited degree the subjects of medical investigation.

Since dental surgery has been professed as a distinct branch of the healing art, there has been a disposition, (and we think a commendable one,) on the part of the medical profession, to confide both the investigation of the principles, as well as the practice of dentistry, to those who make that study and practice their exclusive object. And the Committee would have been quite willing that the diseases incident *to*, and arising *from*, dental practice, should also have been confided to the professors of the dental art, not doubting that they are fully competent to the task, although, from the acknowledged modesty and professional diffidence which characterize *most* of that profession, they might disclaim the possession of the requisite skill. But as the subject has been brought formally before the Medical Society, we observe, that cases have come to our knowledge, through one of the members of this Society, of serious injury resulting to a number of individuals from the use of the royal succedaneum. We have also learned, from other sources, that in many instances bad effects have been produced, and, in some cases, that not only severe and distressing *local* disease has ensued from the use of the mineral paste, but the *lives* of *patients* have been *endangered*. On the other hand, we have the testimony of many persons who have had their teeth filled with the mineral paste, without experiencing any unpleasant effects; on the contrary, the ease, facility, and cheapness of the operation, and the fact that in these instances it has *apparently* answered all the purposes which could have been attained by the use of any other material, and has been used when, in the opinion of *some dentists,* it would have been difficult, perhaps impracticable, to have used gold or tin foil,—all *seems* to imply some value in the practice. The testimony of an experienced dentist,* upon *whose representation of facts* the Committee can rely, is, that within the last two years he has filled about three hundred teeth with the mineral paste, and that, to his knowledge, in no case has the specific effects of mercury been developed. To this statement it has been objected, that in a few instances the effects of mercury upon the mouth, from the use of the mineral paste, have been noticed in this village.

The Committee have no means of ascertaining the proportion of cases in which the mineral paste has produced the specific effects of mercury. As regards the utility of the paste, as a filling for carious teeth, the Committee have examined some cases in which the cavity *seemed* to be accurately filled, some months after the operation. To test this matter, however, by accurate experiment, a small vial was accurately filled with the amalgam, from which the free mercury had been pressed out by the application of great force. The vial was plunged into a colored liquid; in a few hours the fluid was found to have entered the vial by the sides of the amalgam, proving conclusively that the amalgam diminishes in volume in the process of solidification.

That form of the paste which contains the smallest proportion of mercury becomes the hardest. It would *apparently* then, be the object of the dentist to diminish the quantity of mercury to the lowest point necessary to form the amalgam, so that, in the hands of the operator who would give to the article such proportions as would make the most eligible compound for use, the royal succedaneum would be less likely to produce injurious effects.

But it should be observed that the mercury and silver unite in but one proportion, and that any excess of mercury in the compound is in a free state; that when the mixture is subjected to the greatest degree of pressure to remove the free mercury, the amalgam then contains a proportion of sixty-four of mercury to thirty-six of silver.

To account for the activity of so small a portion of mercury, as the ordinary filling of a tooth would contain, the Committee refer to the admitted chemical principle, that "the tendency of

* Dr. S. Bliss.

metals to oxydation is *much* increased by being alloyed." We find this fact so strongly stated and exemplified, by the standard chemical authorities, that the question has arisen in the mind of the committee, whether the presence of an equal quantity of free mercury, would probably be *more pernicious,* than the presence of the amalgam of mercury and silver. On this head the Committee also refer to recent published statements of interesting experiments, in proof of the occasional highly acid state of the saliva, not only in active disease, but in some instances, where there are but slight apparent deviations from health.

If it were admitted that the bad effects of the amalgam have resulted from an undue proportion of mercury, reasoning from the effects of even minute doses of mercury, internally administered, upon some constitutions of peculiar susceptibility to its action, the committee observe, that if the mineral paste containing the largest proportion of mercury, has produced ptyalism, in numerous instances, that it cannot be so combined as not to produce its specific effects in some.

The occasional evil effects of mercury, even when clearly indicated, and used with care and discrimination, have been so often noticed and described by medical practitioners, that the Committee need not enlarge on this point. In most cases, however, in which the local effects of mercury are injurious, some important benefit has been obtained to compensate the patient for his sufferings. If a patient were laboring under hepatitis or syphilis, he would have no good grounds of complaint, if his general malady were relieved, though the local effects of the remedy were severe.

It has been urged, that if the occasional bad effects of mercury used in dentistry, are to preclude its use, by parity of reasoning, it should also preclude its use by the practitioner in all cases. But the difference in the cases arises, not so much from the *nature and effects* of the article in question, as from the relative importance of the object to be attained. To incur the risk of ptyalism for the preservation of a carious tooth, would be one thing—to take the same risk for the probable preservation of life, or for the removal of a serious malady, would be quite another. The first would be a matter of taste, or of probable convenience, the latter would involve the most important consequences.

The Committee enter into no discussion of the modus operandi of mercury in these cases. It would, however, be an interesting subject of inquiry and experiment, whether its peculiar effects are produced by the absorption and *direct* application of the mercurial oxyd to the parts involved, or whether it does pass into the *general circulation*, before its local effects can be evolved. Whether it is possible under any circumstances, and by any management to produce ptyalism, independent of the general circulation.

The Committee have thus endeavored to place before the Medical Society the leading facts in the case, and have added such reflections as naturally arise from the subject. That the mineral paste has produced, in many instances, the peculiar effects of mercury, though in different degrees of intensity, in some slight, in others severe and alarming, there can be no doubt. The Committee believe that the proportion of such cases is small compared with the whole number operated upon, but that no care in the *combination* or *use* of the paste will prevent its occasional bad effects. In view, however, of the *apparently* favorable cases, the question arises in regard to the *probable effects* upon some constitutions from the gradual absorption of the oxydized mercury, even when no *palpable* local effects are induced.

But we leave these points to the consideration of the society, referring the whole subject to it, for such action as it may choose to take, and for the expression of such an opinion of the merits or demerits of the mineral paste, as its regard to the interests of medical science, and its duty to the public may demand.

P. C. Sampson,
Chairman of Committee.

I certify, that at the semi-annual meeting of the Onondaga County Medical Society, held on the 30th January, 1844, at the Temperance House, in the village of Syracuse, the above report was read and unanimously accepted by the Society.

N. R. Tefft,
Secretary.

Few comments are needed upon any portion of this report, and indeed none can be added to strengthen its condemnation of the very article it was expected to eulogize. Unfortunately for mineral paste and its advocates, this committee* was composed of men who early learned to despise quackery, in whatever form it makes its appearance—whose love for truth and science, led them to investigate, and when conclusions were fairly arrived at, they were not by the naked assertion of a single man, though old in years, to be deterred from giving the whole truth. This report will have a most salutary influence on the community whence it originated, and will do honor to the able gentlemen by whom it was made. Although as I have stated, nothing can be added to give it strength, it contains several suggestions, which have not been fully discussed, and to which I propose to give some further notice.

The first is in relation to the testimony of individuals. The report under this head, reads as follows, viz. "On the other hand we have the testimony of many persons who have had their teeth filled with the mercurial paste, without experiencing any unpleasant effects."

In respect to such testimony, we remark, that it is very rarely to be trusted, especially when the effect is gradual, and the individual is not put to extreme inconvenience, or positive suffering; since this subject has been under discussion, I have met in several instances with persons who complained of an extraordinary flow of saliva, both by day and by night, but who could hardly be made to believe it was caused by several large amalgam fillings. And even before this effect is produced, this mercurial action is pervading the system, only waiting for some developing circumstances to bring out its marked and specific character, in the form of salivation. How often do we meet with the most extensive disease in the mouth, such as inflamed and ulcerated gums, alveolar abcesses, teeth loosened by tartar, &c., while the persons positively declare, that their teeth and gums are entirely well, and to prove conclusively their position, they assert that "they never ache." Actual pain is made a standard of *disease*, and any thing short of this, I may say, is *generally* regarded a proof of health.

Now salivation considered as a disease, it may be remarked,

* P. C. Sampson, M. D.; M. M. White, M. D.; E. T. Richardson, M. D., Committee.

unless very intense, never produces pain. It may, therefore, be safely assumed that the specific effects of mercury may not only be carried to the extent of vitiating the fluids of the mouth, but actual salivation might occur without the individual suspecting, in the least, the origin of the difficulty.

Is any other proof demanded of the uncertainty of such testimony, either in respect to disease or remedies, we may refer to the massive testimony of marvellous cures by quack nostrums, with which our newspapers are daily loaded—whereas the truth is, that very few who thus testify, know any thing either as to the nature of the disease treated, or the remedy given, or indeed what medicine they had been taking.

The next point is in relation to the testimony of "the dentist" who uses this paste.

The following is the language of the report:

"The testimony of an experienced dentist,* upon whose representation of facts, the committee can rely, is, that within the last two years, he has filled about three-hundred teeth with the mercurial paste, and that to his knowledge, in no case has the specific effects of mercury been produced."

In respect to such testimony, it would seem, unexplained, somewhat surprising. For a time this astonishing result was accounted for by this "experienced dentist," by saying, "he pressed out all the free mercury through a rag."

But since it has been clearly shown that the mercury actually combined in the silver, was *more* likely to become oxydized than *free* mercury, I have not heard his amendment to this *rag*ged explanation. Without spending time in conjecturing what would be the next turn, I will offer what, in case "his representation of facts *can* be relied on," must be the real solution of the mystery. We will suppose these cement patients divided into two classes, the one embracing all on whom the specific action of mercury had not amounted to severe ptyalism, and the other embracing those whose suffering had compelled them to seek relief. The former class, as has been shown, not recognising any special difficulty, would apply to no one for consultation or advice, and this class I freely admit, would embrace much the greater proportion.

But what of the others—

* Dr. Bliss.

That fox which had lost in a trap, an important member, would by no means be distinguished for his cunning, should he apply for relief to him who set and sprung the trap. Nor would a salivated patient sooner apply to a dentist, *however "experienced"* he might be, to be restored from a disease which his culpable *ignorance* or neglect had brought upon him. Hence, *if at all*, he could say of a truth, that in no instance had any evil result followed *such* operations. That many of the cases which did not come to this "experienced dentist's" *knowledge*, came to the knowledge of this *committee* is certain, and formed no small item in bringing them to the conclusions found in this report.

The fact that this paste, by whomsoever made, unless it contain one of the ingredients in a free state, must be of the same composition, though an important one, has perhaps been sufficiently commented upon by the Committee. The attractions of this amalgam based on its *apparent* success, "the ease, facility and cheapness" have been also fully discussed in the remarks preceding this report.

I shall only add to the foregoing facts and consideration, the report of the Committee appointed by the American Society of Dental Surgeons at their last meeting, to collect facts to lay before the Medical Committee of Onondaga County.

This report is as follows:

The Committee appointed by the American Society of Dental Surgeons, at its Annual Meeting in Baltimore, July, 1843, to which was referred the subject of metallic paste, or amalgam, used by certain individuals for stopping carious teeth, begs leave to report. That at the aforesaid meeting of the above named society, it was, "*Unanimously Resolved*, That this society regards the use of mineral pastes, as stoppings for carious teeth, as malpractice."

The Committee, furthermore, refers to a circular issued in May, 1841, in the city of New York, relating to the identity of the material employed by a certain Mons. Mallan, and that used a few years before by the notorious Messrs. Crawcour, in which it is proved by the unquestionable testimony of Dr. Chilton, a distinguished chemist of the aforesaid city, that mercury and silver were the ingredients of the paste used by the persons above named, by which so many fine teeth were wholly and irretrievably destroyed.

It may not be irrelevant to state, that such was the indignation of the public sentiment, in regard to the use of this amalgam by the Crawcours, that the officers of justice pursued the ship in which they had precipitately embarked for Europe, as far as the Hook, in a steamboat chartered for the purpose, with the intention of bringing them to the punishment which their flagrant imposture so richly deserved, but the captain of the ship did not find it convenient to permit the officers to go on board, and thus the renegades succeeded in making their escape.

In regard to the departure of Mons. Mallan, after a short visit to our country, the Committee asks leave to refer to a notice of that fact given in the first number of the fourth volume of the American Journal and Library of Dental Science, issued Sept. 1843.

"*The last Importation of Trans-atlantic Quackery re-shipped for England, with the usual allowance of 'drawback.'*

"The famous, or more properly the *infamous*, Mons. Mallan, of mineral paste notoriety, the worthy successor of the Messieurs Crawcour, has recently taken French leave of his tailor, his shoemaker, his upholsterer, his printers, 'et id omne genus,' who have allowed him to run up bills without footing them, during the past two years, in the city of New York. The profession throughout the country are indebted to some of their enterprising brethren of that city, for having made such an *expose* of the malpractices of this consummate quack, as to induce the good people of New York to be on their guard in relation to employing a foreign mountebank, instead of the honorable members of the dental profession who are located among them.

"It will be remembered by most of our subscribers, that a circular was forwarded to them about two years ago, containing extracts from English journals, together with affidavits taken in New York, with respect to the practices and professions of the individual named above. Such was the effect of this circular as to prevent the empirical vagabond from gaining a footing in any other place than New York, where he was reduced to the alternative of either flight or starvation.

"Such is the salutary effect of associated effort in suppressing imposition in dental practice. We hope our brethren, in all parts of the country, will exhibit equal enterprise, in every similar case; to the end that the empirics of the old world may learn to expect no success in the new. The editors of some of the London journals deserve our thanks for warning the American public of the approach of the above named charlatan to our shores; and we hereby reciprocate the favor, by informing them that he has lately returned to their island."

The Committee states, furthermore, that according to the best knowledge and belief of all the honorable members of the profession with whom the individuals composing this Committee have conversed, the mineral paste, in use among the quacks and charlatans of the dental profession in the United States and Great Britain, is this identical amalgam of mercury and silver, which can be inserted firmly in a tooth by mere pretenders, who find it impossible to make a permanent stopping with a proper material.

Among a host of examples of the destructive properties of this amalgam, when inserted in human teeth, which have come to the knowledge of every dentist in extensive practice, the Committee asks leave to detail the following case, which occurred in the practice of the chairman of this Committee, on the 30th ult., at No. 1 Bond street, in the city of New York:

A lady of Belleville, in the state of New Jersey, called on Dr. E. Parmly, on the day above-named, in great distress, from disease in the first right molar tooth of the upper jaw. On examining the mouth, he found the gum around the aforesaid tooth to be of a dark hue, and nearly or quite destitute of healthy circulation. The tooth itself was also dark and necrose, and evidently demanding immediate extraction. Before removing the tooth the operator passed a probe along the side of the tooth, completely into the cavity of the antrum maxillare. When the tooth was extracted, profuse hæmorrhage succeeded, and considerable portions of ash-colored bone adhered slightly to the fangs. Six or eight other pieces of bone, about the size of small peas, were afterwards removed from the cavity of the sinus. The cavity was filled with blood, which made its escape through the nostrils, down the throat, and out of the mouth of the patient. The lady informed Mr. Parmly that she had been often nearly strangled at night by purulent matter, which made its escape from the antrum through the nostrils into the fauces, larynx, and trachea. The operator found so large an opening into the antrum, after removing the diseased tooth and decomposed bone, that he could easily pass his little finger quite up to the sub-orbital parietes of the cavity. This tooth had been filled with a very large plug of mercury and silver, which was unquestionably the cause of the deplorable effects already described.

The Committee therefore expresses the unqualified opinion, that mineral paste is wholly unsuitable for the purpose of filling the cavities of carious teeth, and should never be employed for that purpose. In confirmation of the opinion which the Committee have thus expressed, they subjoin the following certificates.

Eleazer Parmly,
Solyman Brown,
Joseph H. Foster, M. D.

On the twentieth day of July, 1843, I was called to visit a lady who was suffering from a tooth filled with the mineral amalgam. This lady had been sent to my office nearly a fortnight previous, by her physician, to have the same tooth removed. Not remembering the number, she stopped at the first door in the place, and inquired of a gentleman standing there if he knew the number of my house. He could not or would not give her the desired information, but observing her face to be bandaged, inquired what operation she intended to have performed. She told him, extraction. He then remarked, "there is no necessity, madam, for you to lose your tooth, come into my office, and I will fill it for you." She accordingly consented, and underwent the operation, and paid him his fee of five dollars. When it was finished, she told him that the tooth was aching more violently than before. "Give yourself no uneasiness, madam, the pain will be of short duration, and you will then have no more suffering, and your tooth will be useful to you for a long time." That ignorant, foreign mountebank and charlatan, Mons. Mallan, who so effectually succeeded in imposing upon the credulity of many of our citizens, is the individual to whom I am giving full credit for the operation, and the consequent effects of it in this case.

For a week succeeding the operation, this patient suffered the most acute agony, passing sleepless nights, and obtaining only occasional relief by the use of opiates, and declining to send for her physician because she did not like to inform him that she had acted contrary to his directions. On examination, I found the periosteum of the inferior maxillary bone much inflamed, the adjacent soft parts tumefied, very hard, and acutely sensitive to the touch. The interior of the mouth was in an equally inflamed state, and the patient had been for several days, and was still, severely salivated. With some difficulty, I succeeded in extracting the tooth, the second molaris; on the side next to the dens sapientiæ, I found a large cavity, and in and around it, a filling which I knew to be the same composition which Dr. Chilton had analyzed, as before used by Mons. Mallan, viz. a compound of mercury and silver. Removing the filling, I found the chamber of the tooth and the interior of the fangs very dark colored, and the confined matter highly offensive. The cavity had apparently been filled without any attempt to remove the diseased portion. An excavating instrument had not been used, but the filling was pressed into the cavity upon the exposed nerve, and not only into the cavity, but into the natural space between the teeth, and under the gums, and from being thus in direct contact with the mucous membrane of the mouth, it had acted upon the glandular system so as to produce active ptyalism. Dr. Wooster informed me that this lady had previously enjoyed good health, and that he had not administered any mercurial medicines.

I could cite many other cases which have come under my own observation, in which this article, called by as many assumed names as there are different operators who use it, has been productive of much serious injury; one such case, however, is sufficient to convince any one who may witness it, what I have been compelled to believe, and now assert, viz. That no individual in the profession, who places any value upon his own reputation, or who expects or deserves to retain the confidence which those who seek his services repose in him, will incur the risk of using this mineral amalgam. Those who do use it are not likely to know the extent of the injury which they do, for when serious suffering results, patients are not apt to return to the cause of all their trouble to seek relief, but go elsewhere for advice.

JOSEPH H. FOSTER, M. D.

I hereby certify, that the facts, as stated by Dr. Foster, in relation to the above case, are correct; that I was the constant medical attendant of the lady referred to, and know her to have been a comparatively strong and healthy person previous to this event; that I had had no necessity for, and had not administered any mercurial medicine previous to the extraction of the tooth, and that on my first visit after the operation by Mons. Mallan, I found her glandular system had been affected so as to cause active mercurial salivation. I continued my attendance upon her for four months afterwards, during the whole of which time she was unable to attend to her family duties. The swelling of the face could not be reduced by any remedies, and continued the cause of so much acute suffering, that a fever ensued, and very active treatment was resorted to at one period to prevent a threatened inflammation of the brain. Exfoliation of the inferior maxillary bone I was constantly apprehensive of, until I became convinced that the case had assumed the form of osteo sarcoma.

At the end of four months, when this lady left me to go to the south, her face had not resumed its proportions, nor could I perceive any approximation to a change, and her pain still continued.

I have no doubt that all her sufferings are justly attributable to the mineral amalgam so much used in filling teeth, and which, I am convinced, is not only an improper, but a highly dangerous compound. JOSEPH WOOSTER, M. D.

### ARTICLE VIII.

*The Dental Surgeon Defined.* By C. T. CUSHMAN, Columbus, Ga.

